# Supplementary material for: Development and validation of two economical and flexible immunoassays for detecting antibodies against LCMV in mouse serum
Source: bioRxiv. 2026 Apr 8:2026.04.07.716918. Preprint. [Version 1] doi: 10.64898/2026.04.07.716918 (PMC13081961; doi:10.64898/2026.04.07.716918)
Supplement: Supplement 1 [file NIHPP2026.04.07.716918v1-supplement-1.pdf]

bioRxiv preprint doi: https://doi.org/10.64898/2026.04.07.716918; this version posted April 8, 2026. The copyright holder for this preprint (which was not certified by peer review) is the author/funder, who has granted bioRxiv a license to display the preprint in perpetuity. It is made available under aCC-BY 4.0 International license.

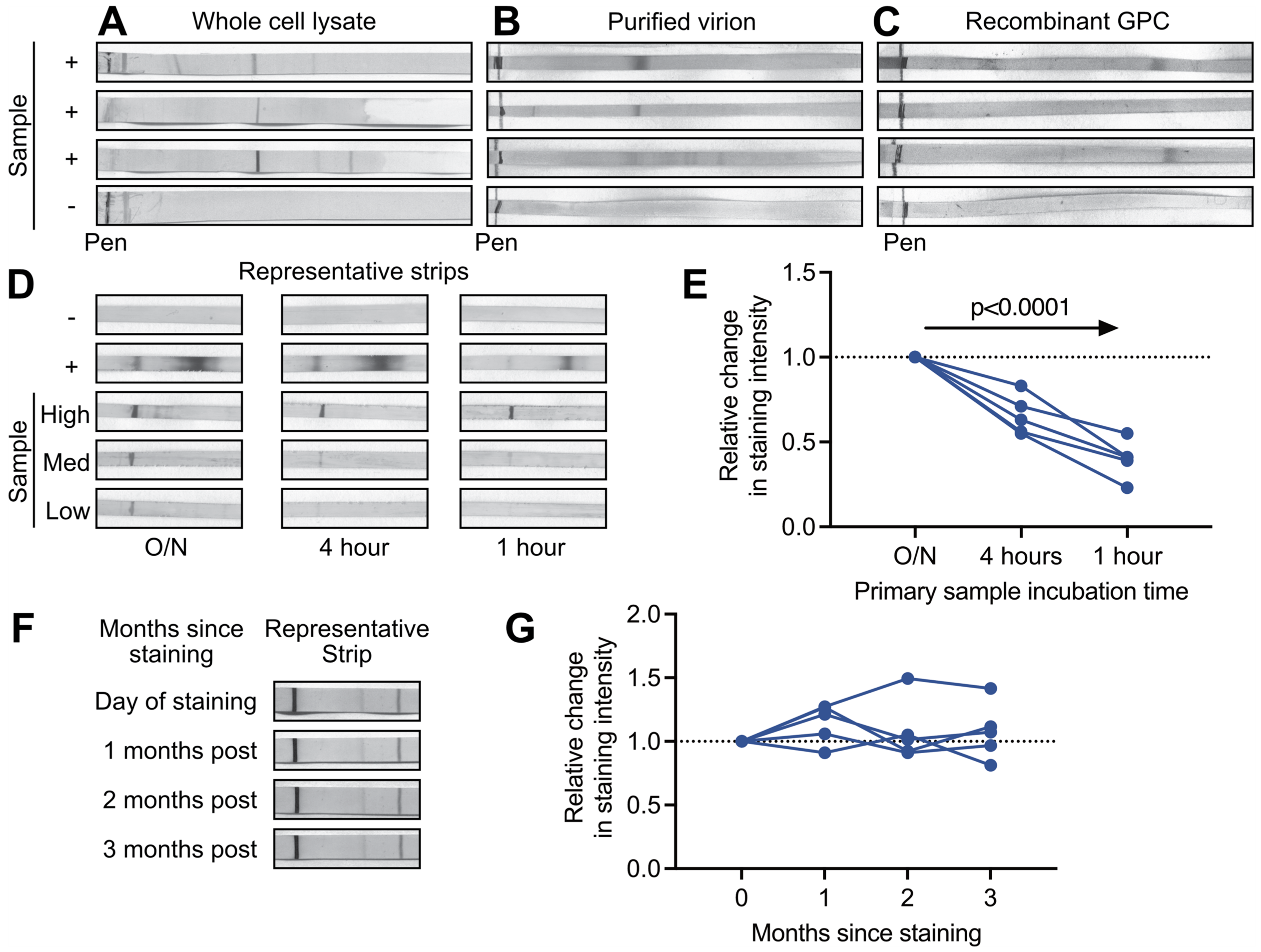

Supplemental Fig 1. Testing of strip immunoblot conditions. (A-C) Comparison of expected results from western blot membrane strips containing either (A) whole cell lysate from LCMV Armstrong 53b-infected BHK cells at 48 hpi, (B) sucrose banded LCMV Armstrong 53b virions derived from Vero E6 cells, or (C) purified recombinant glycoprotein (GPC) (aa 266 to 498 of strain Armstrong 53b). In each case, strips were exposed to serum from an uninfected, seronegative mouse (-) or from mice infected at 8 weeks of age and sampled at 28 dpi (n=3 independent samples). (D-E) Results of blocking duration optimization SIA testing with (D) showing representative staining for overnight, 4 hours, and 1 hour blocking incubations while (E) quantifies results as connected absolute value data points per sample. Positive control staining was done with 33.6, a known antibody specific for the LCMV glycoprotein (33.6, GPC/GP2) used at 1:500. SIA test in (D) used serum samples with a known high, medium, and low endpoint titer from previous CBA tests. (F-G) Colorfast test of SIA staining with representative strips from day of staining, plus 2, 4, and 6 months-post staining. (F) Quantification of (E) compared to intensity of same band from day of staining using average grey pixels measure in ImageJ. Data represented in (E, G) as connected absolute values for each sample. Statistical comparisons made in (E, G) as a one-way ANOVA test for linear trend. All quantification of staining intensity assessed using the average grey pixels measure in ImageJ.

bioRxiv preprint doi: https://doi.org/10.64898/2026.04.07.716918; this version posted April 8, 2026. The copyright holder for this preprint (which was not certified by peer review) is the author/funder, who has granted bioRxiv a license to display the preprint in perpetuity. It is made available under aCC-BY 4.0 International license.

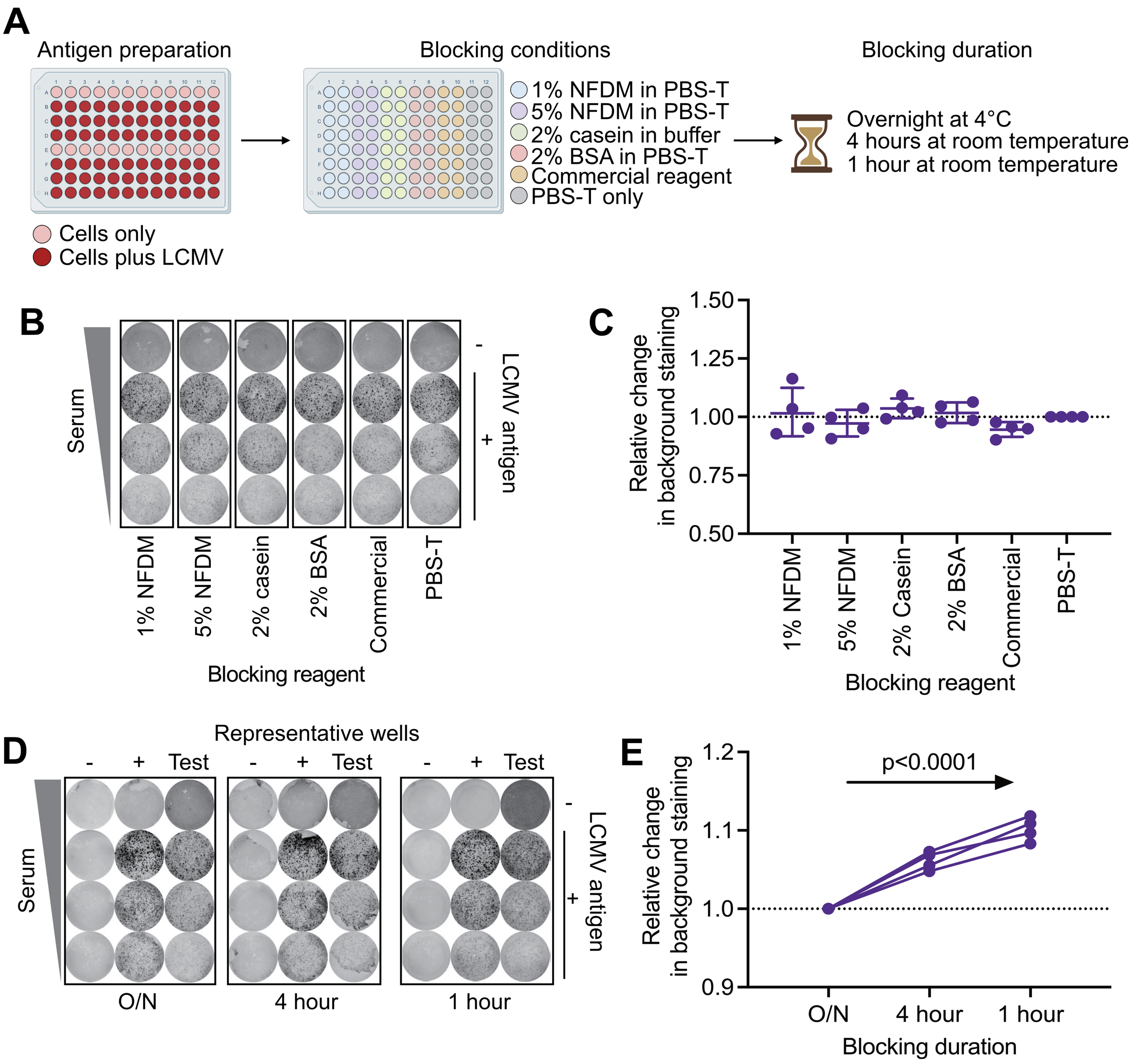

483

484 **Supplemental Fig 2. Optimization of cytoblot immunoassay.** (A) Test of blocking conditions
485 for CBA. (B) Representative comparison of blocking reagents in CBA using serum from a known
486 positive mouse. (C) Quantification of relative staining antigen negative wells in (B) represented
487 as means ± SEM. (D-E) Results of blocking duration for CBA with (D) showing representative
488 staining for overnight, 4 hour, and 1 hour blocking while (E) quantifies results as connected
489 absolute values data points per sample. Positive control staining is a known LCMV antibody for
490 the nucleoprotein (1-1-3, 1:1000 starting dilution). Negative control staining is phosphate
491 buffered saline. Data represented in (C) as individual datapoints and in (F) as connected
492 absolute values for each sample. Statistical comparisons made in (C) using one-way ANOVA
493 with Dunnett's multiple comparisons test setting PBS-T as control and in (E) as a one-way
494 ANOVA test for linear trend. All quantification of staining intensity assessed using the average
495 grey pixels measure in ImageJ. Schematic in (A) designed with bioRender.

bioRxiv preprint doi: https://doi.org/10.64898/2026.04.07.716918; this version posted April 8, 2026. The copyright holder for this preprint (which was not certified by peer review) is the author/funder, who has granted bioRxiv a license to display the preprint in perpetuity. It is made available under aCC-BY 4.0 International license.

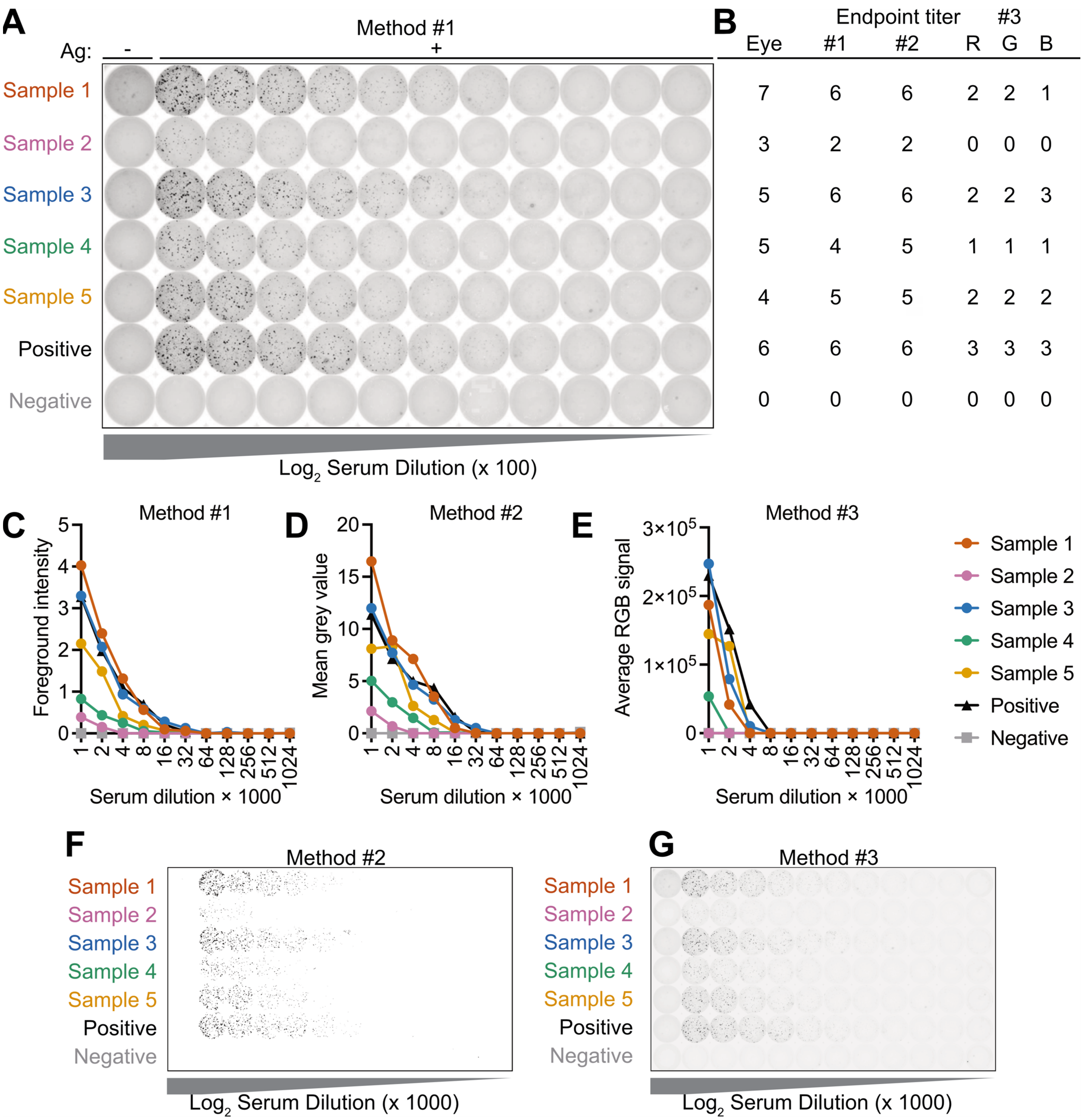

Supplemental Fig 3. Comparison of seropositivity and endpoint titer calculation methods. (A) Stained CBA plate with representative samples showing a variety of endpoint titers. (B) Comparison of endpoint titer calculation by eye or with automated methods. (C-E) Quantification of staining as represented by images in (A), (F), or (G), respectively. (C) Method #1 calculates the relative intensity of identified foreground objects in each region of interest in (A). (D) Method #2 calculates total black pixels in a region of interest in (F). (E) Method #3 separates the image data in (G) into red, green, and blue channels which are then quantified by the software. Data in (C-E) is graphed as absolute values for each sample. Positive control is staining with a known LCMV antibody (1.1.3, 1:1,000 starting dilution). Negative control staining is phosphate buffered saline.

bioRxiv preprint doi: https://doi.org/10.64898/2026.04.07.716918; this version posted April 8, 2026. The copyright holder for this preprint (which was not certified by peer review) is the author/funder, who has granted bioRxiv a license to display the preprint in perpetuity. It is made available under aCC-BY 4.0 International license.

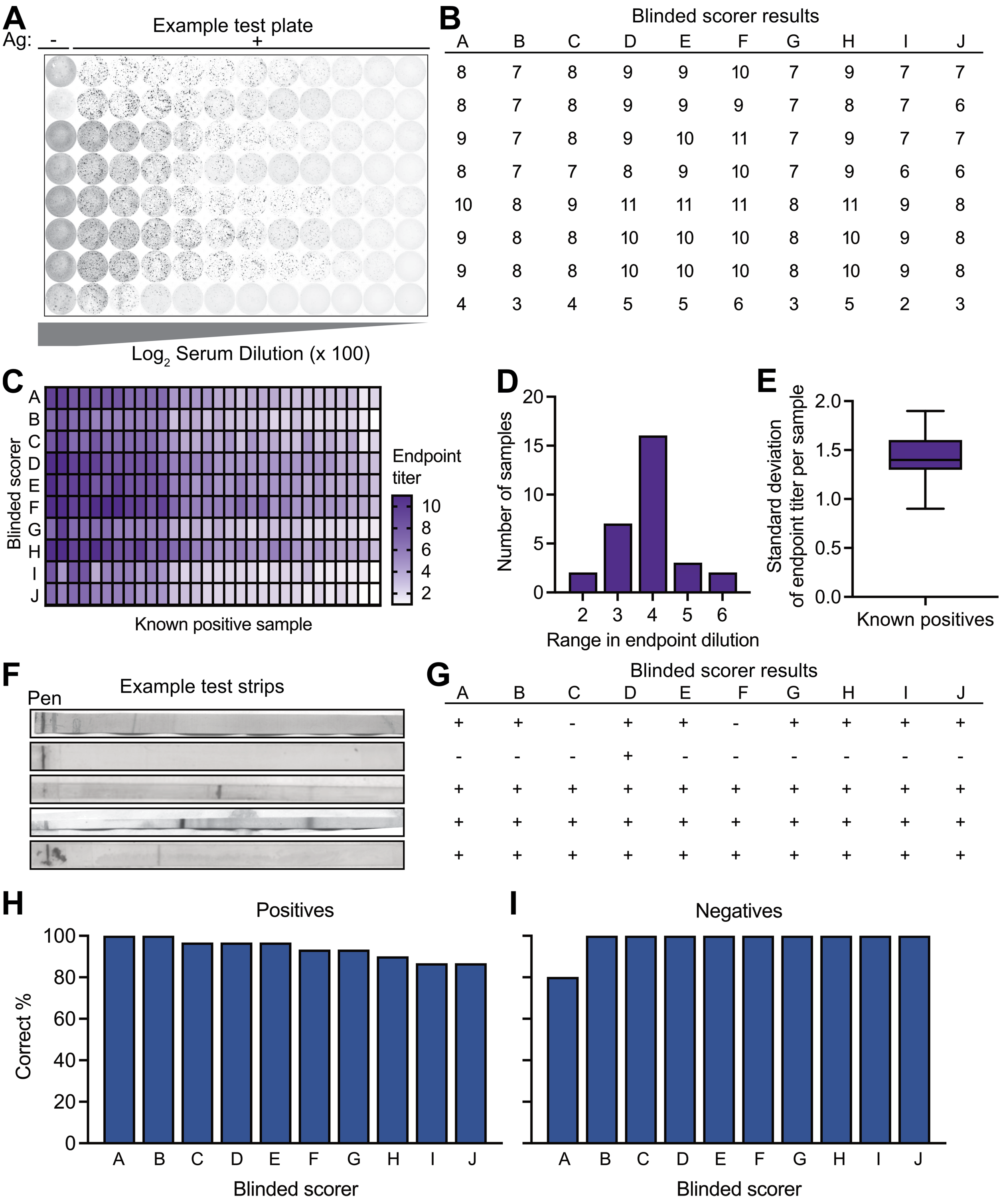

Supplemental Fig 4. Testing of assay accuracy in trained individuals. (A) Example of test CBA plate provided to trained scorers with (B) individual raw results. (C-E) Variability of endpoint titer determination across 30 known positive samples in the CBA with (D) representing the range in endpoint titer and (E) indicating the standard deviation of each sample endpoint titer. (F) Example of n=5 test SIA stripblots provided to trained scorers with (G) individual raw

bioRxiv preprint doi: https://doi.org/10.64898/2026.04.07.716918; this version posted April 8, 2026. The copyright holder for this preprint (which was not certified by peer review) is the author/funder, who has granted bioRxiv a license to display the preprint in perpetuity. It is made available under aCC-BY 4.0 International license.

results. (H, I) Percentage of known (H) positive and (I) negative samples correctly identified by each blinded scorer. Data graphed in (D) as a histogram of range in endpoint titer for each positive sample, in (E) as box plots with whiskers indicating the minimum to maximum values and the center line indicating median values and in (H, I) as percent correct. Trained scorers (n=10) were blinded to sample identification prior to assessment and did not participate in the completion of the assays. Each individual scorer is designated with a letter-labeled column in panels (B), (G), and (H-I).
